# Regulation of Dietary Protein Solubility Improves Ruminal Nitrogen Metabolism In Vitro: Role of Bacteria–Protozoa Interactions

**DOI:** 10.3390/nu14142972

**Published:** 2022-07-20

**Authors:** Zhenbin Zhang, Wenjun Wei, Sihan Yang, Zeliang Huang, Chuang Li, Xiang Yu, Ruxin Qi, Wujun Liu, Juan J. Loor, Mengzhi Wang, Xin Zhang

**Affiliations:** 1 College of Animal Science and Technology, Yangzhou University, Yangzhou 225009, China; 18762304859@163.com (Z.Z.); weiwennjunn@163.com (W.W.); maomaozhu2022@163.com (S.Y.); hzl1015486489@gmail.com (Z.H.); chuangliyzu@163.com (C.L.); yyxx970826@163.com (X.Y.); ruxinqi@126.com (R.Q.); 2 State Key Laboratory of Sheep Genetic Improvement and Healthy Production, Xinjiang Academy of Agricultural Reclamation Sciences, Shihezi 832000, China; 3 College of Animal Science, Xinjiang Agricultural University, Urumqi 830001, China; wujunliu1026@xjau.edu.cn; 4 Mammalian Nutrition Physiology Genomics, Department of Animal Sciences and Division of Nutritional Sciences, University of Illinois, Urbana, IL 61801, USA; loor@illinois.edu

**Keywords:** dietary interventions, soluble protein, nitrogen metabolism, rumen bacteria, ciliate protozoa

## Abstract

Precision dietary interventions (e.g., altering proportions of dietary protein fractions) has significant implications for the efficiency of nutrient use in ruminants, as well as lowering their environmental footprint, specifically nitrogen (N) emissions. Soluble protein (SP) is defined as the protein fraction that is rapidly degraded in the rumen (e.g., non-protein N and true protein), and our previous study found that regulating SP levels could improve N efficiency in *Hu* sheep. Thus, the present study was conducted to explore in vitro how protein fractions with different SP levels modulate the rumen microbial community and its association with N metabolism. Four dietary treatments with different SP proportions and similar crude protein (CP) content (~14%) were formulated (% of CP): 20 (S20), 30 (S30), 40 (S40) and 50 (S50). Results showed that NH_3_-N content increased with increasing SP levels at 4, 12 and 24 h; TVFA, acetate, propionate and valerate were higher in S30 and S40 (*p* < 0.05) and had quadratic effects (*p* < 0.05). Moreover, dry matter digestibility (DMD) and N digestibility (ND) were all decreased with S20 and S50 (*p* < 0.05). The S30 and S40 treatments increased the abundance of Bacteroidetes and *Prevotella* (*Prevotella_ruminicola*) but decreased the abundance of Firmicutes and Proteobacteria (*p* < 0.05). Bacterial pathways related to amino acid and fatty acid metabolism also were enriched with S30 and S40. The abundance of *Entodinium* was increased with S30 and S40 and had a positive correlation with *Prevotella*, and these two genera also played an important role in N metabolism and VFA synthesis of this study. In conclusion, bacterial and protozoal communities were altered by the level of SP (% of CP), with higher SP levels (~50% of CP) increasing the microbial diversity but being detrimental to rumen N metabolism.

## 1. Introduction

With the rapid development of the modern economy, the demand for high-protein food sources is increasing year by year, and beef, mutton, and dairy products (especially ruminant products) seem to be the first choice for most consumers [1,2]. However, ruminant production is one of the main drivers of global environmental degradation, and the contribution to environmental pollution is much greater compared with non-ruminants [3,4]. Ruminants account for over 70% of global livestock ammonia (NH_3_) emissions and around 30% of total anthropogenic NH_3_ emissions, resulting in significant economic losses and negative human health impacts [5,6]. In China, 55–59% of total nitrogen (N) emissions in the past decade (2004–2014) have been emitted into the air in gaseous form, with agricultural and livestock production accounting for the largest amount (62–69%) [7]. Hence, reducing the environmental impact of N emissions while meeting the growing demand for ruminant products is one of the main goals of current sustainable food production systems.

Feed nutrient composition is considered an important reference strategy to improve N retention and reduce N emission in ruminant production, e.g., using low-protein diets [8] or adjusting the rumen degradable to non-degradable protein ratio with a non-soluble to soluble carbohydrates ratio in diets [9]. Our previous study on *Hu* sheep indicated that when reducing crude protein (CP) content as well as adjusting soluble protein (SP) to 25–30%, N efficiency was increased [10]. Stefanski et al., isolated different protein fractions from rapeseed meal and demonstrated a different efficiency of ruminal N metabolism of these fractions in vivo [11]. Undeniably, feed protein fractions play an important role in nutrient absorption of ruminants, specifically the efficiency of N utilization.

Current research on SP in ruminant production has been mainly focused on establishing models of rumen metabolism, typically fractions PA1 (non-protein nitrogen, NPN) and PA2 (true protein) described by the latest CNCPS version 6.5 [12]. However, diet-microbiome interactions in the context of SP are rarely reported. Our previous research indicated that altering dietary SP levels to ~25–30% with decreasing CP increased the relative abundance of Prevotellaceae and *Prevotella_1* in the rumen [10]. Ciliate protozoa accounting for an estimated 25% to 50% of microbial biomass in the rumen are also involved in the breakdown of nitrogenous substances, especially insoluble proteins [13]. Despite the antagonism of predation in protozoa–bacteria interactions, positive interactions (e.g., metabolic exchange) have also been demonstrated [14,15]. For instance, ciliate protozoa convert a small amount of insoluble protein that cannot be used in the rumen into SP for bacterial growth and utilization, thus contributing to dietary protein degradation [16]. Solomon et al., reported the existence of protozoa-mediated enrichment of the Gammaproteobacteria family and genera and increased the overall diversity of the genera *Prevotella* and *Treponema*, which resulted in significant individual variation in end-product metabolites including methane [17].

Rumen bacteria–bacteria interactions have attracted considerable attention in recent years, and our previous work has investigated the relationship between rumen bacteria, metabolites and phenotype when feeding *Hu* sheep low-protein diets with different SP. However, there is a paucity of information on how bacteria–protozoa interactions play a role in the rumen degradation of SP. In this study, we aimed to evaluate rumen bacterial communities, protozoal communities, and bacteria–protozoa interactions, as well as their associations with N metabolism in response to different SP levels in vitro. We hypothesized that altering SP levels could regulate rumen bacterial and protozoal communities, which result in different efficiency of N metabolism in vitro.

## 2. Materials and Method

### 2.1. Ethical Statement

All of the procedures performed on the animals followed the Guidelines of the Animal Welfare Committee of Yangzhou Veterinarians of the Agriculture Ministry of China under approved permit number SYXK (Su) 2021-0026.

### 2.2. Substrate Preparation and Determination of Protein Fractions

The substrate components consisted of six feeds, including rice straw, corn, soybean meal, wheat bran, corn protein meal and urea. The rice straw was harvested after maturity in the autumn of 2020 from the experimental farm of Yangzhou University. It was then dried naturally in the field. The other five concentrates were purchased through local commercial suppliers. All feeds were sampled by quartering and drying at 65 °C to a constant weight, and then they were ground to pass through a screen (1 mm) by a Retsch ZM 100 Wiley mill (Retsch GmbH, Haan, Germany) and stored. The SP content of six feeds was determined by following buffer-soluble N protocols in the standardization of procedures for N fractionation [18], which was described concretely in our previous study [10], and specific protein fractions (CP and SP values) are reported in Table S1.

### 2.3. Animal Management and Experimental Design

Ruminal fluid used for the experiment was selected from 3 male *Hu* sheep (body weight 59.7 ± 4.8 kg; age 18 ± 1 months) with permanent ruminal fistulas (Yangzhou University). Animals were raised in single pens and equipped with automatic drinking devices and feed troughs. The diet was fed (dry matter (DM) basis) to approximately 3% body weight, and water was available for ad libitum consumption. The diet was a total mixed diet consisting of 60% rice straw, 30% corn, 6% soybean meal, 2.5% wheat bran, and 1.5% premix, with daily feeding at 7:00 and 19:00 in equal amounts.

The feed ingredients described in Section 2.2 were used to configure the four dietary treatments, which consisted of a fixed amount of rice straw (50%) combined with concentrates in order to achieve the different SP proportions (as % of CP) of 20, 30, 40 and 50%, namely S20, S30, S40 and S50, respectively. The CP content of the substrates in each group was relatively consistent (~14%), and the modification in SP (% of CP) was mainly achieved by manipulation of the ingredient’s inclusion in the concentrate portion. The substrate composition and nutrient levels are shown in Table 1.

### 2.4. Rumen Fluid Inoculation and In Vitro Fermentation

Methods of ruminal fluid collection and in vitro culture referred to the protocols of our previous publication [19]. Briefly, a vacuum plastic catheter (internal diameter: 10 mm) was used to collect rumen fluid from three different points (vestibule, rucksack, abdominal sac) in the rumen before morning feeding. The rumen fluid filtrate was mixed and put into a thermos bottle (preheated to 39 °C and filled with CO_2_ to ensure anaerobic conditions) after filtering through four layers of gauze, then the bottle cap was immediately closed and quickly returned to the laboratory. Artificial buffer solution was prepared according to the method of Menke and Steingass [20] in which the chemical reagent of buffer solution was dissolved in 1 L of distilled water and included NaHCO_3_, NH_4_HCO_3_, Na_2_HPO_4_, KH_2_PO_4_, MgSO_4_·7H_2_O, Na_2_S, MnCl_2_·4H_2_O, CoCl_2_·6H_2_O, FeCl_3_·6H_2_O, CaCl_2_·2H_2_O and resazurin. Before inoculation, buffer solution was preheated in a water bath oscillator (SHA-A, Hengfeng Instrument, Jintan, China) at 39 °C, and high-purity CO_2_ was continuously introduced to adjust the pH to 6.8 until the solution was colorless. Then, the filtered rumen fluid and buffer solution were thoroughly mixed at a volume ratio of 1:2 under anaerobic conditions. A total of 60 mL of the mixed solution was used as a culture medium to dispense into a 200 mL fermentation flask with substrate (1.0000 ± 0.1000 g), shaken and placed at 39 °C for a 24 h incubation.

A total of 120 fermentation flasks (4 treatments × 5 sampling times × 6 replicates each time) were used and sampled at different time points for the determination of fermentation parameters, substrate digestibility and microbial communities (bacteria and protozoa).

### 2.5. Sample Collection and Analysis

The fermentation flasks were taken out quickly at 2, 4, 8, 12, and 24 h, and placed on ice to terminate the fermentation. In total, 5 mL of each culture medium was collected, and the pH value was measured immediately with a pH meter (pHS-3C, Shanghai, China). Ammonia N (NH_3_-N) concentration was measured according to Broderick and Kang (phenol-hypochlorite system) [21], microbial protein (MCP) content was determined by the purine prediction method [22]; gas chromatography (GC-14B, Kyoto, Japan) was used for determination of volatile fatty acids (VFAs), with crotonic acid as the internal standard [23], and specific procedure settings referred to our previous publication [10]. The substrate residues at each time point were collected to detect dry matter digestibility (DMD) and N digestibility (ND). The residue of the substrate was filtered through a nylon bag (20 μm pore size), then dried at 65 °C to constant weight, and the DMD was finally calculated by the weight of the disappearance. ND at 24 h was determined and calculated via a Kjeldahl analyzer (FoodALYT D4000, Bremen, Germany).

### 2.6. DNA Extraction, Library Construction, 16S/18S rRNA Sequencing and Data Processing

The fecal genomic DNA extraction kit (DP328-02, Tiangen Biochemical Technology Co., Ltd., Beijing, China) was used to extract the total genomic DNA of culture media samples at 24 h. DNA concentration and purity were monitored on 1% agarose gels. According to the concentration, DNA was diluted to 1 ng/μL with sterile water. 16S/18S rRNA genes in distinct regions were amplified with specific primer (e.g., 16S V3–V4: 341F(5′-CCTAYGGGRBGCASCAG-3′)-806R(5′-GGACTACNNGGGTATCTAAT-3′), 18S V4: 316F (5′-GCTTTCGWTGGTAGTGTATT-3′)-539R (5′-CTTGCCCTCYAATCGTWCT-3′)) and barcodes [24]. All PCR mixtures contained 15 μL of Phusion^®^ High-Fidelity PCR Master Mix (New England Biolabs), 0.2 μM of each primer and 10 ng target DNA, and cycling conditions consisted of a first denaturation step at 98 °C for 1 min, followed by 30 cycles at 98 °C (10 s), 50 °C (30 s) and 72 °C (30 s) and a final 5 min extension at 72 °C. An equal volume of 1× loading buffer (contained SYB green) was mixed with PCR products and electrophoresis was performed on a 2% agarose gel for DNA detection. The PCR products were mixed in equal proportions, and then Qiagen Gel Extraction Kit (Qiagen, Germany) was used to purify the mixed PCR products.

Following the manufacturer’s recommendations (Novogene Co., Ltd., Tianjin, China), sequencing libraries were generated with NEBNext^®^ Ultra™ IIDNA Library Prep Kit (Cat No. E7645). The library quality was evaluated on the Qubit@ 2.0 Fluorometer (Thermo Scientific, Wilmington, DE, USA) and Agilent Bioanalyzer 2100 system. Lastly, the library was sequenced on an Illumina NovaSeq platform and 250 bp paired-end reads were generated. Paired-end reads were merged using FLASH (Version 1.2.11) [25], whereas quality filtering on the raw tags was performed using the fastp (Version 0.20.0) software [26] to obtain high-quality Clean Tags. The Clean Tags were compared with the reference database (Silva database https://www.arbsilva.de/ for 16S/18S, (accessed on 11 February 2022)) using Vsearch (Version 2.15.0) to detect the chimera sequences, and then the chimera sequences were removed to obtain the Effective Tags [27]. Denoise was performed with the DADA2 module in the QIIME2 software (Version 202006) to obtain initial ASVs (Amplicon Sequence Variants), and then ASVs with an abundance less than 5 were filtered out [28]. Species annotation was performed using QIIME2 based on the Silva Database, and the relative abundance of 7 classification levels (domain, phylum, class, order, family, genus and species) was obtained. Alpha diversity (Chao1 and Shannon index) and beta diversity (PCoA) based on the Bray–Curtis dissimilarity matrix were calculated in QIIME2 and visualized via the “ggplot2 package” in an R program (v 3.6.1). The Wilcoxon rank-sum test was used to analyze differences between treatments at the phylum and genus levels for bacteria and protozoa and visualized via “ggtree” and “ggplot2” packages.

Bacterial function predictions were performed via the PICRUSt 2 package (https://github.com/picrust/picrust2/wiki, (accessed on 11 February 2022)) [29] with pathways annotated in the Kyoto Encyclopedia of Genes and Genomes (KEGG) database. Principal component analysis (PCA) was performed on the absolute abundance results of pathway annotation. Linear discriminant analysis (LDA) effect size (LEfSe) was used to compare the marker pathways among SP treatments (LDA score > 2.5, *p* < 0.05).

### 2.7. Statistical Analysis

Data for pH, NH3-N, MCP and DMD were analyzed using PROC MIXED of SAS software (version 9.3, SAS Institute Inc., Cary, NC, USA), and analytical models included fixed effects (treatment, sampling time, treatment × sampling time) and random effects (sample replicates). PROC GLM procedure was performed for NH_3_-N, VFAs, ND, bacterial/protozoal alpha diversity and species level of bacteria. Tukey’s method was applied for multiple comparisons among treatments. Linear and quadratic effects were tested by orthogonal polynomial contrasts, with significance declared at *p* ≤ 0.05 and tendency at 0.05 < *p* ≤ 0.1. Spearman’s rank correlation analysis was performed for the interaction of rumen bacteria, protozoa and fermentation, and the rank coefficient and *q* value (FDR-adjusted *p*-value) were calculated via the “Psych” package in an R program, with the threshold declared at *q* < 0.05 or |r| > 0.5. The correlation heatmaps were visualized by the “ggcorrplot” R package.

## 3. Results

### 3.1. In Vitro Rumen Fermentation and Digestibility

Table 2 shows that the sampling time and the interaction between time and treatment had significant effects on pH value (*p* < 0.05), which decreased over time. However, there was no difference among treatments at different sampling times (*p* > 0.05).

The sampling time and SP treatments had significant effects on NH_3_-N content (Table 3, *p* < 0.05), which showed first a decrease and then an increase. S50 were all highest at 4, 12 and 24 h compared with other treatments (*p* < 0.05).

Since significant differences among SP treatments were observed above, trend analysis (Table S2) at each time point was performed, NH_3_-N content showed significant linear and quadratic effects at 4 h (*p* < 0.05), while at 12 and 24 h, NH_3_-N content linearly increased with increasing substrate SP (*p* < 0.05).

Table 4 showed that sampling time had significant effects on MCP content (*p* < 0.05), which fluctuated irregularly with time, while SP treatments and the interaction between time and treatment had no significant effects (*p* > 0.05).

As shown in Table 5, VFA concentrations differed with TVFA, acetate and propionate, being higher in S30 and S40 (*p* < 0.05), while valerate was increased in S30 compared with S20 and S50 (*p* < 0.05). With the increase in SP (% of CP), they showed quadratic effects (*p* < 0.05).

**Figure 1:**
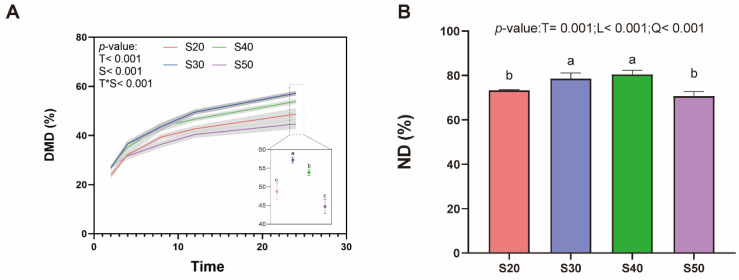
Substrate digestibility under different SP treatments (n = 6). (**A**) Substrate dry matter digestibility (DMD) changes at different sampling times, different letters indicate significant differences among SP treatments at *p* < 0.05. T: treatment; S: sampling time; T × S: interaction between T and S. (**B**) Substrate nitrogen digestibility (ND) at 24 h of culture. Bar charts with different letters indicate a significant difference at *p* < 0.05. Treatments: S20, S30, S40 and S50 are ~14% CP, SP proportion (% of CP) 20, 30, 40 and 50, respectively. T: treatment; L: linear; Q: quadratic.

Sampling time and SP treatments had significant effects on DMD (Figure 1A), and S30 was the highest at 24 h compared with the other three treatments (*p* < 0.05), while as shown in Figure 1B, ND in S30 and S40 was higher than S20 and S50 at 24 h (*p* < 0.05).

### 3.2. Bacterial Diversity and Taxonomic Differences In Vitro

**Figure 2:**
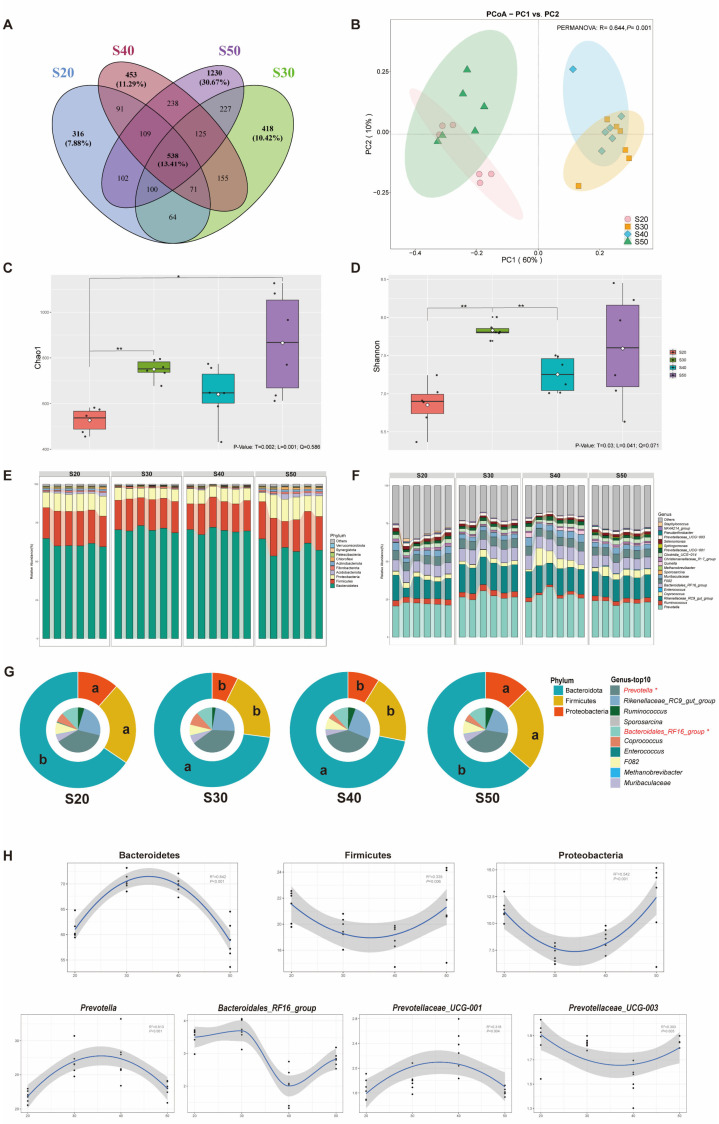
Bacterial diversity, taxonomic differences and changing tendency (n = 6). (**A**) Venn diagram showing shared and unique bacterial OTU numbers of four SP treatments. (**B**) Principal coordinate analysis (PCoA) of bacteria based on Bray–Curtis dissimilarity matrix of four SP treatments. (**C**,**D**) Alpha diversity of bacteria in four SP treatments, * represents *p* < 0.05 and ** represents *p* < 0.01. Bacterial compositions of four SP treatments at the phylum (**E**) and genus (**F**) levels. (**G**) Pie chart of differential microbes in four SP treatments, different letters indicate significant difference of SP treatments at *p* < differential microbes in four SP treatments, different letters indicate significant difference of SP treatments at *p* < 0.05. (**H**) The trend of differential microbes changing with SP. Treatments: S20, S30, S40 and S50 are ~14% CP, SP proportion (% of CP) 20, 30, 40 and 50, respectively.

A total of 2,598,686 raw reads were obtained from in vitro rumen samples for bacterial 16S rRNA genes, and 51,654 effective tags on average were achieved after quality control. Lastly, 5,010 ASVs were generated from all samples. A Venn diagram (Figure 2A) revealed that the intersection ASVs of four treatments were 538, which accounted for 13.41% of the total ASVs, the numbers of unique ASVs for the S20, S30, S40 and S50 were 316 (7.88%), 418 (10.42%), 453 (11.29%), and 1230 (30.67%), respectively. The PCoA results (Figure 2B) revealed significant differences in the bacterial communities (PERMANOVA: *p* = 0.001). In alpha diversity (Figure 2C,D), the Chao 1 index with the S30 and S50 was higher than S20 (*p* < 0.05), while the Shannon index with S30 was higher than S20 and S40 (*p* < 0.05).

Bacteroidetes, Firmicutes and Proteobacteria were the dominant phyla (Figure 2E) and *Prevotella*, *Rikenellaceae_RC9_gut_group* and *Ruminococcus* were the dominant genus (Figure 2F), multiple comparisons (Figure 2G) found that S30 and S40 increased the abundance of Bacteroidetes and *Prevotella* but decreased the abundance of Firmicutes and Proteobacteria (*p* < 0.05); meanwhile, they showed quadratic effects (Figure 2H, *p* < 0.05). At the species level (Table 6), *Prevotella_ruminicola* was increased in S30 and S40 (*p* < 0.05), S50 increased *Fibrobacter_succinogenes* compared with S30, and *bacterium_AC2043* in S40 was lower than S20 and S50 (*p*< 0.05).

### 3.3. Predicted Ruminal Microbial Functions via PICRUSt2

**Figure 3:**
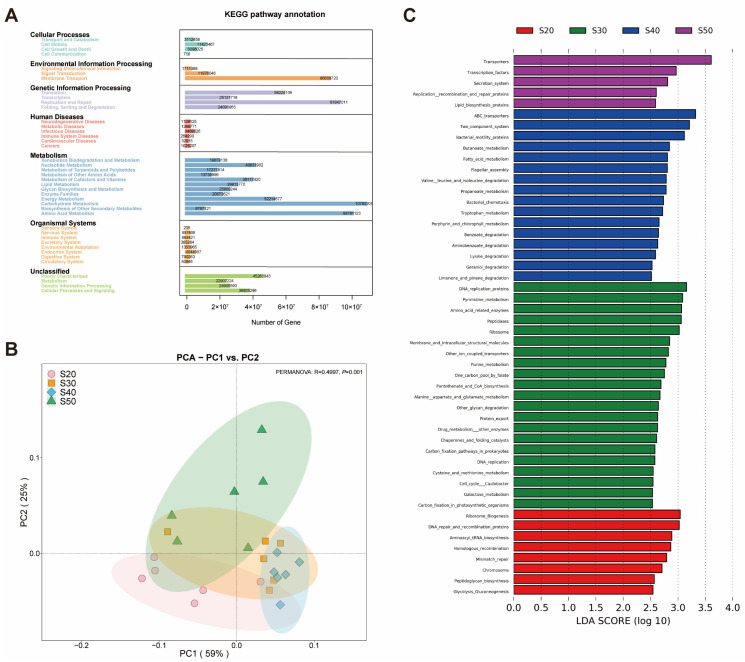
Bacterial function prediction based on the KEGG database via PICRUSt2. (**A**) Statistical histogram of KEGG pathway prediction results based on the 16S gene copy number. (**B**) Principal Component Analysis (PCA) of absolute abundance of functional annotations based on the KEGG database. (**C**) Linear discriminant analysis (LDA) plus effect size of function prediction, LDA score ≥ 2.5 with *p* < 0.05 was marked. Treatments: S20, S30, S40 and S50 are ~14% CP, SP proportion (% of CP) 20, 30, 40 and 50, respectively.

KEGG pathway annotation results (Figure 3A) showed that the main functions of bacterial genes were concentrated in metabolism, genetic information processing and environmental information processing (level 1), especially carbohydrate metabolism, amino acid metabolism and energy metabolism (level 2). PCA results (Figure 3B) showed significant differences in bacterial gene function among SP treatments (PERMANOVA: *p* = 0.001). At level 3 metabolic pathways (Figure 3C), we found many functions were enriched on S30 and S40 (LDA > 2.5, *p* < 0.05), especially carbohydrate metabolism and amino acid metabolism: amino-acid-related enzymes (S30); alanine, aspartate, and glutamate metabolism (S30); cysteine and methionine metabolism (S30); galactose metabolism (S30); lysine degradation (S40); valine, leucine and isoleucine degradation (S40); propanoate metabolism (S40); and butanoate metabolism (S40).

### 3.4. Protozoal Diversity and Taxonomic Differences In Vitro

**Figure 4:**
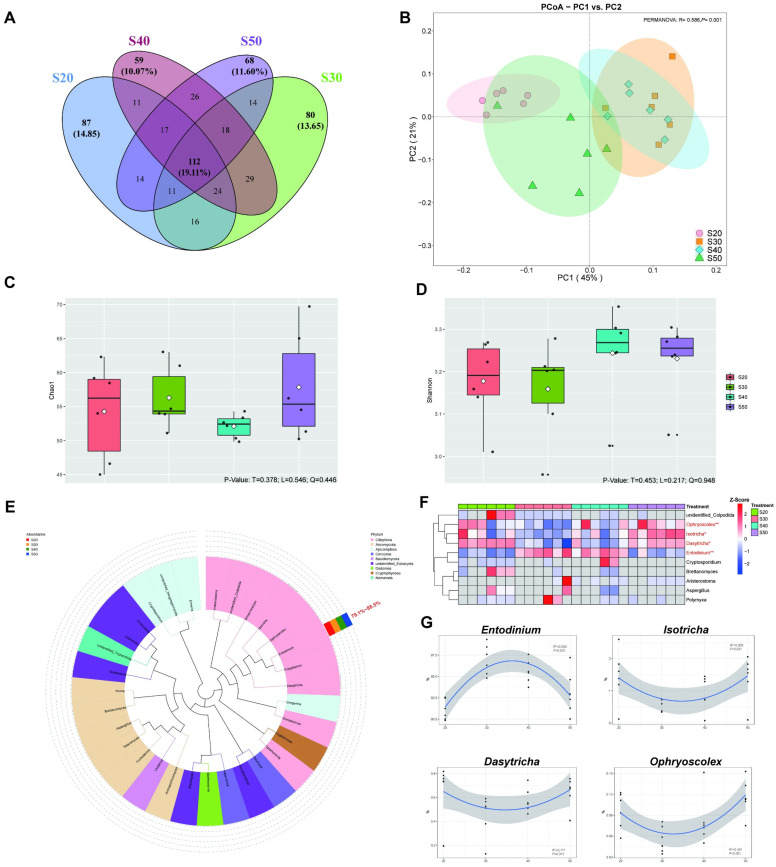
Protozoa composition diversity, taxonomic differences and changing tendency (n = 6). (**A**) Venn diagram showing shared and unique protozoal OTU numbers of four SP treatments. (**B**) Principal coordinate analysis (PCoA) of protozoa based on Bray–Curtis dissimilarity matrix of four SP treatments. (**C**,**D**) Alpha diversity of protozoa in four SP treatments. (**E**) Protozoa genus evolutionary tree, different colors represent the classification phylum level. (**F**) Heatmap of the top-10 protozoa genera in relative abundance, * represents *p* < 0.05 and ** represents *p* < 0.01. (**G**) The trend of differential protozoa changing with SP. Treatments: S20, S30, S40 and S50 are ~14% CP, SP proportion (% of CP) 20, 30, 40 and 50, respectively.

In total, 2,724,311 raw tags were derived from the protozoa 18S rRNA gene sequencing, with 84,079 effective tags on average achieved from all samples after quality control. Based on the effective tags of all samples, OTUs (Operational Taxonomic Units) clustering with 97% identity resulted in 590 OTUs. A Venn diagram (Figure 4A) revealed that the intersection OTUs of four treatments was 112 (19.11% of total), and the numbers of unique OTUs for the S20, S30, S40 and S50 were 87 (14.85%), 80 (13.65%), 59 (10.07%), and 68 (11.60%), respectively. The PCoA results (Figure 4B) revealed significant differences in the protozoal communities among SP treatments (PERMANOVA: *p* = 0.001). However, no difference was found in protozoal alpha diversity (Figure 4C,D).

Thirty-one taxa were identified at the genus level and had an evolutionary relationship between genera and taxonomic phyla (Figure 4E), which mainly belong to Ciliophora (>99%). Entodinium was the dominant genus, which accounted for 79.1%~88.5% (relative abundance), followed by Isotricha (1.2%~2.5%). By comparing the four treatments (Figure 4F), S30 and S40 increased the abundance of Entodinium but decreased the abundance of Dasytricha (*p* < 0.05), which also had quadratic effects (Figure 4H, *p* < 0.05). In addition, S50 up-regulated the abundance of *Isotricha* and *Ophryoscolex* compared with S30 and S40 (*p* < 0.05).

### 3.5. The Interaction of Rumen Bacteria, Protozoa and Fermentation

**Figure 5:**
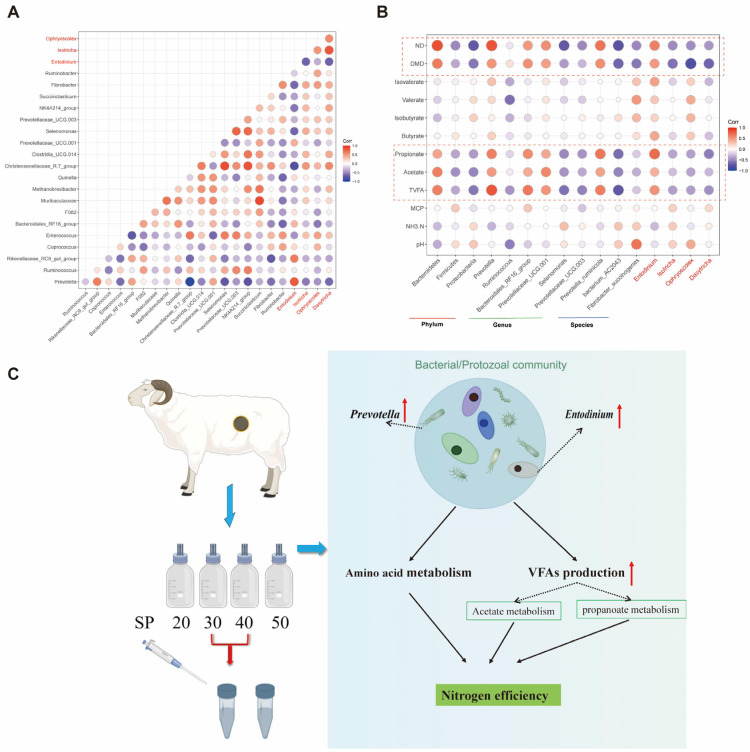
The interaction of rumen bacteria, protozoa and fermentation. (**A**) Spearman’s rank correlations between rumen bacteria and protozoa at the genus level. (**B**) Spearman’s correlations between rumen microbiota and fermentation. Based on the color key on the right, the degree of each correlation is displayed in the shade of the color; the blue circle represents negative correlations, and the red circle represents positive correlations. The red fonts represent the protozoa genus. (**C**) Integrative diagram showing the regulatory role of bacteria–protozoa interactions in rumen nitrogen metabolism as SP (% of CP) set as 30 or 40 in vitro.

Spearman’s rank correlations between rumen bacteria genus and protozoa genus showed that *Prevotella* had strong positive correlations with *Entodinium*, while negative correlations with *Isotricha, Ostracodinium* and *Dasytricha* (Figure 5A). On the other hand, different rumen microbiota had strong correlations with TVFA, acetate, propionate, DMD and ND, especially Bacteroidetes, *Prevotella, Prevotella_ruminicola* and *Entodinium*, which had strong positive correlations with them (Figure 5B).

## 4. Discussion

Regulation of dietary protein fractions is one of the important nutritional intervention strategies to improve nutritional metabolism in ruminants. Our previous in vivo experiments have shown that SP levels (% of CP) of ∼25–30% have higher N efficiency with dietary CP content decreased by∼10% in *Hu* sheep [10]. In this study, as time progressed, the pH value of each treatment gradually decreased and fluctuated from 6.85 to 5.90, which was mainly due to the decomposition and utilization of substrate nutrients by rumen microorganisms and the accumulation of organic acids [30]. It is worth noting that the urea addition level of the substrate gradually increased (0~1.6% DM). Tian et al. [31] showed that the hydrolysis of urea is alkaline; thus, with the increase in urea addition, the pH value also increased. However, the change in pH was not obvious among treatments in this study, which may be related to the urea addition levels.

The fluctuation of NH_3_-N concentration in the rumen reflects the degradation of dietary N and the utilization of NH_3_-N by rumen microorganisms. Generally, the optimal NH_3_-N concentration of rumen microorganisms is 6.3–27.5 mg/dL [32]. In our study, the NH_3_-N concentration of each treatment fluctuated from 9.6 to 24.5 mg/dL, which was within the normal range. However, SP treatments significantly changed the NH_3_-N concentration, especially in the S50, in which it was at a high level from 4 h of in vitro fermentation, which may be mainly related to the higher SP level (50% of CP) in the substrate. A large amount of SP in the substrate was rapidly decomposed in the rumen to generate ammonia, polypeptides and amino acids, while excess ammonia could not be utilized by rumen microorganisms [33]. The above results were also consistent with the in vivo study. Similarly, Wilson et al. [34] reported that the NH_3_-N concentration in the rumen was significantly up-regulated with increasing dietary SP (% of CP) from 34.4% to 44.9%. At 24 h, as the substrate SP (% of CP) was 30, the NH_3_-N concentration was lower, which may reflect the balance of substrate carbon and N degradation, and the rumen microorganisms could utilize the generated NH_3_-N to a greater extent.

Stern et al. [35] found that substrate protein solubility (22.7 vs. 36% of CP) had no significant effect on MCP synthesis in vitro, and high SP substrates (36% of CP) seemed to reduce the DM digestibility, which was similar to our results of decreased DM digestibility in S50, the reason might be that the release of substrate energy and N in S50 was asynchronous, which led to the decline of fermentation efficiency [36]. Moreover, Hume et al. [37] reported that the maximum synthesis of MCP was related to the NH_3_-N concentration (8.8 mg/dL), which is comparable to the concentration in the S30 at 8 h (10.93 mg/dL), and the MCP content at this time point was indeed the highest numerically. In addition, our previous in vivo study also found that with the increase in SP levels (21.2–29.4%), no difference was found in MCP content.

From the view of the rumen microbiome, the abundance of Bacteroidetes was improved, and Firmicutes and Proteobacteria were decreased as SP (% of CP) was 30 and 40. The total relative abundance of these three bacterial phyla in this study was over 80%. The host digests plant feed primarily through fermentation mediated by the microorganisms in these three phyla levels [38]. In addition, VFA, acetate, propionate and valerate were also elevated with S30 and S40, which may be attributed to the enrichment of *Prevotella*, which was the core genus of Bacteroidetes. The functions of this microbe include the fermentation of carbohydrates, the utilization of nitrogenous substances, and the biotransformation of bile acids and other steroids; the main by-products of anaerobic respiration are acetate, isovalerate, and succinate, which are closely related to VFA biosynthesis [39,40]. Moreover, propionate is a major substrate of gluconeogenesis, and a recent study also showed that it is closely related to ruminal N metabolism [41]. Unsurprisingly, bacteria functional annotations were also enriched in fatty acid metabolism and propanoate metabolism (Figure 3C), which was consistent with our findings in vivo.

*Prevotella_ruminicola* (*P. ruminicola*) plays an important role in the metabolism of proteins and peptides in the rumen [42]. *P. ruminicola* has the greatest range and activity of dipeptidyl peptidases, which release dipeptides from the N-terminus of peptides [43] and may be the main reason for the higher substrate N digestibility in S30 and S40 and also the strong positive correlation of this microbe with ND (Figure 5B). A recent study [44] showed that lower methane emissions and higher feed conversion efficiency were associated with a higher abundance of *P. ruminicola*, which was also validated from the results that substrates DMD and ND were increased in S30 and S40, while methane production requires subsequent experiments to verify. Furthermore, it was observed that the abundance of *Fibrobacter_succinogenes* (*F. succinogenes*), which is considered to be a key cellulolytic bacterium that promotes the metabolism of other microbiota members by degrading cellulose into soluble sugars and succinate [45], was up-regulated in S30 and S40. In spite of the weak peptidolytic activity, studies have reported that when *P. ruminicola* was co-cultured with *F. succinogenes*, the substrate nutrition digestibility was increased compared with culturing *F. succinogenes* alone [46]. In addition, the bacterial functions were enriched in related amino acid metabolism and carbohydrate metabolism at SP (% of CP) of 30 and 40, which also verified the higher substrate nutrient utilization obtained in S30 and S40 in our study.

Similar to most studies on rumen protozoa [47,48], *Entodinium* was the most abundant genus of ciliate protozoa, occupying an average of 83.8% in this study. Polyorach et al. [49] and Chanjula et al. [50] found that different dietary N sources had no effect on the number of ruminal protozoa, which may lead to different protozoa species composition. In our study, SP levels were modified by the addition of different proportions of N, while *Entodinium* was up-regulated when SP (% of CP) was 30 and 40. *Entodinium* could rapidly hydrolyze true protein (e.g., casein) and has high dipeptidase activity, with peptides and amino acids as main products [51,52]. However, it has been proposed that urea plays a minor role in the N metabolism of *Entodinium* when used as a substrate [53], which indicates that the change in the addition of urea as a substrate in our study might not be the reason for affecting the abundance of *Entodinium*. On the other hand, the abundance of *Isotricha* and *Ophryoscolex* was reduced with S30 and S40, and the species members in this group of microbes exhibit lower proteolytic activity [54]. Although the abundance of these protozoa genera was relatively lower, more experiments are required to reveal the association with SP levels in the future.

The interaction of bacteria and protozoa plays an important role in the degradation of substrates in this experiment, with *Prevotella* and *Entodinium* being strongly and positively correlated (Figure 5A). Similarly, Elsayed Mickdam et al. [55] reported that the relative abundance of *Prevotella* and *Entodinium* was elevated in the presence of feed-induced subacute ruminal acidosis in vitro, which might be related to lower ruminal pH, while *Prevotella* could be used as a probiotic to prevent acidosis [56]. Importantly, the synergistic effect of the two genera resulted in higher N efficiency of substrates, while Prevotella was negatively correlated with *Isotricha* and *Ophryoscolex*, which may be a result of phagocytosis of protozoa and bacteria or the autolysis of protozoa [57].

Last but not least, a limitation of this study is that the diets were not configured with constant soluble carbohydrates content, which could potentially affect the N retention of the ruminal SP. Different soluble carbohydrate levels may exacerbate differences between treatments, such as NH_3_-N production and MCP synthesis. Therefore, the consistency of soluble carbohydrates needs to be refined in subsequent experiments by configuring the SP gradient.

## 5. Conclusions

Regulating substrate SP levels could modify bacterial and protozoal communities in vitro, especially the abundance of *Prevotella* and *Entodinium*, which were up-regulated as SP (% of CP) was 30 or 40 and ultimately led to higher nutrient utilization efficiency (e.g., N efficiency). Similar to our previous in vivo study, NH_3_-N concentration was increased with increasing SP levels. Furthermore, TVFA, acetate, propionate and valerate all increased with S30 and S40, and bacterial pathways were also enriched in related amino acid and fatty acid metabolism (Figure 5C). Overall, these results enhance our knowledge of the role of dietary SP level on the regulation of rumen microbiome and N efficiency. Although higher SP levels (~50% of CP) increased microbial diversity, they were detrimental to rumen N metabolism, underscoring the importance of the balance of carbon and N degradation in the rumen.

## Figures and Tables

**Table 1:** Substrate composition and nutrient levels.

Item	Treatments			
	S20	S30	S40	S50
Ingredient, %				
Rice straw	50	50	50	50
Corn	35	34	33	33.4
Soybean meal	5	6.5	6.9	1
Wheat bran	4	6.45	9.25	14
Corn protein meal	6	2.65	-	-
Urea	-	0.4	0.85	1.6
Total	100	100	100	100
Nutritive level, g/kg				
DM	888.75	887.53	886.63	886.86
CP	137.91	137.22	137.89	138.93
SP (% of CP)	20.43	30.12	39.95	49.50
EE	43.00	43.10	43.26	42.08
Ash	65.75	66.27	66.85	65.86
NDF	350.23	358.3	366.62	380.67
ADF	199.88	198.86	199.06	205.46
Ca	1.02	1.02	1.03	0.98
P	2.44	2.47	2.53	2.58

DM, dry matter; CP, crude protein; SP, soluble protein; EE, ether extract; NDF, neutral detergent fiber; ADF, acid detergent fiber; Ca, calcium; P, phosphorus. Treatments: S20, S30, S40 and S50 are ~14% CP, SP proportions (% of CP) 20, 30, 40 and 50, respectively.

**Table 2:** The effect of substrate SP (% of CP) levels on pH value at different sampling times in vitro.

Sampling Time/h	Treatment				SEM	*p*-Value		
	S20	S30	S40	S50		S	T	S × T
2	6.77	6.78	6.74	6.76	0.004	<0.001	0.408	<0.001
4	6.67	6.63	6.68	6.65				
8	6.55	6.54	6.56	6.52				
12	6.32	6.33	6.35	6.46				
24	5.90	5.94	6.00	6.02				

Treatments: S20, S30, S40 and S50 are ~14% CP, SP proportion (% of CP) 20, 30, 40 and 50, respectively; SEM: standard error of the mean; S: sampling time; T: treatment; S × T: interaction between S and T.

**Table 3:** The effect of substrate SP (% of CP) levels on NH_3_-N content at different sampling times in vitro (mg/100 mL).

Sampling Time/h	Treatment				SEM	*p*-Value		
	S20	S30	S40	S50		S	T	S × T
2	18.65	17.52	17.21	19.24	0.257	0.002	0.002	0.037
4	11.79 ^b^	12.17 ^b^	12.66 ^b^	20.96 ^a^				
8	11.82	10.93	12.65	13.78				
12	11.67 ^c^	12.26 ^c^	14.48 ^b^	19.16 ^a^				
24	15.61 ^c^	15.85 ^c^	16.20 ^bc^	20.45 ^a^				

^a–c^ values with different superscripts differ significantly at *p* ≤ 0.05 among SP treatments. Treatments: S20, S30, S40 and S50 are ~14% CP, SP proportion (% of CP) 20, 30, 40 and 50, respectively; SEM: standard error of the mean; S: sampling time; T: treatment; S × T: interaction between S and T.

**Table 4:** The effect of substrate SP (% of CP) levels on MCP content at different sampling times in vitro (mg/mL).

Sampling Time/h	Treatment				SEM	*p*-Value		
	S20	S30	S40	S50		S	T	S × T
2	1.40	1.37	1.36	1.26	0.043	<0.001	0.258	0.884
4	1.39	1.23	1.46	1.75				
8	1.67	1.74	1.67	1.72				
12	1.76	1.58	1.79	1.65				
24	2.15	1.76	1.84	2.21				

Treatments: S20, S30, S40 and S50 are ~14% CP, SP proportion (% of CP) 20, 30, 40 and 50, respectively; SEM: standard error of the mean; S: sampling time; T: treatment; S × T: interaction between S and T.

**Table 5:** The effect of substrate SP (% of CP) levels on volatile fatty acid (VFA) concentration at 24 h in vitro.

Item	Treatment				SEM	*p*-Value		
	S20	S30	S40	S50		T	L	Q
TVFA, mM	78.67 ^b^	91.69 ^a^	89.30 ^a^	80.04 ^b^	2.065	0.031	0.050	0.017
Acetate, mM	46.50 ^b^	53.88 ^a^	52.66 ^a^	47.58 ^b^	1.205	0.022	0.015	0.005
Propionate, mM	20.76 ^b^	24.56 ^a^	23.46 ^a^	21.19 ^b^	0.565	0.030	0.181	0.036
Butyrate, mM	9.36	10.58	10.39	9.88	0.259	0.253	0.149	0.067
Isovalerate, mM	0.84	0.98	0.95	0.85	0.023	0.712	0.650	0.369
Valerate, mM	0.69 ^b^	0.81 ^a^	0.78 ^ab^	0.70 ^b^	0.019	0.033	0.223	0.045
Isobutyrate, mM	0.53	0.59	0.55	0.53	0.011	0.411	0.333	0.182
A/P	2.24	2.19	2.25	2.24	0.009	0.108	0.373	0.187

A/P: Acetate: Propionate; ^a,b^ values with different superscripts differ significantly at *p* ≤ 0.05 among SP treatments. Treatments: S20, S30, S40 and S50 are ~14% CP, SP proportion (% of CP) 20, 30, 40 and 50, respectively; SEM: standard error of the mean; T: treatment; L: linear; Q: quadratic.

**Table 6:** The effect of substrate SP (% of CP) levels on the relative abundance of bacterial species at 24 h of culture in vitro (%).

Phylum	Genus	Species	Treatment				SEM	*p*-Value		
			S20	S30	S40	S50		T	L	Q
Firmicutes	*Coprococcus*	*Rumen_bacterium*	4.23	2.99	6.88	2.99	0.852	0.359	0.984	0.446
Bacteroidota	*Rikenellaceae_RC9_gut_group*	*Bacteroidales_bacterium*	3.81	3.70	5.32	3.11	0.392	0.236	0.885	0.178
Bacteroidota	*Prevotella*	*Prevotella_ruminicola*	1.48 ^b^	1.83 ^a^	1.95 ^a^	1.50 ^b^	0.079	0.044	0.756	0.008
Bacteroidota	*Bacteroidales_RF16_group*	*Rumen_bacterium*	1.55	1.62	1.69	1.55	0.057	0.825	0.901	0.417
Firmicutes	*Selenomonas*	*Selenomonas_ruminantium*	1.11	1.01	0.79	1.21	0.078	0.281	0.919	0.110
Proteobacteria	*Ruminobacter*	*Ruminobacter_amylophilus*	0.45	0.38	0.39	0.80	0.101	0.445	0.267	0.266
Fibrobacterota	*Fibrobacter*	*Fibrobacter_succinogenes*	0.51 ^ab^	0.72 ^a^	0.52 ^ab^	0.31 ^b^	0.054	0.034	0.038	0.031
Firmicutes	*Selenomonas*	*Rumen_bacterium*	0.55	0.43	0.39	0.50	0.030	0.185	0.438	0.048
Bacteroidota	*Rikenellaceae_RC9_gut_group*	*Rumen_bacterium*	0.25	0.28	0.38	0.31	0.021	0.165	0.135	0.237
Firmicutes	*Christensenellaceae_R-7_group*	*Bacterium_AC2043*	0.12 ^a^	0.07 ^ab^	0.03 ^b^	0.13 ^a^	0.014	0.006	0.961	0.001

^a,b^ values with different superscripts differ significantly at *p* ≤ 0.05 among SP treatments. Treatments: S20, S30, S40 and S50 are ~14% CP, SP proportion (% of CP) 20, 30, 40 and 50, respectively; SEM: standard error of the mean; T: treatment; L: linear; Q: quadratic.

## Data Availability

The sequencing data were deposited in the National Center for Biotechnology Information (NCBI) under BioProject accession number: PRJNA780247 (BioSample ID SAMN28162086).

## Supplementary Materials

The following supporting information can be downloaded at: https://www.mdpi.com/article/10.3390/nu14142972/s1, Table S1: Protein fractions of the feed samples; Table S2: Trend analysis of each treatment’s NH_3_-N content at different time points in vitro.
