# Supplementary material for: Regulation of Dietary Protein Solubility Improves Ruminal Nitrogen Metabolism In Vitro: Role of Bacteria–Protozoa Interactions
Source: Nutrients. 2022 Jul 20;14(14):2972. doi: 10.3390/nu14142972 (PMC9325197; doi:10.3390/nu14142972)
Supplement: Supplementary file 1 [file nutrients-14-02972-s001.zip › nutrients-1812744-supplementary.pdf]

Table S1. Protein fractions of the feed samples.

Items	Protein fractions (Mean, n=3)		
	CP[1] %	SP[2] (%CP)	ISP[3] (%CP)
Rice straw	8.2	20.4	79.6
Corn	8	24.3	75.7
Soybean meal	46.9	35.9	64.1
Wheat bran	15.4	33.7	66.3
Corn protein meal	65.5	6.4	93.6
Urea	281	100	0

[1]CP = crude protein;

[2]SP = soluble protein;

[3]ISP = insoluble protein.

Table S2. Trend analysis of each treatment's NH_3-N content at different time points *in vitro*.

Sampling Time/h	Treatment				SEM	*P*-Value		
	**S20**	**S30**	**S40**	**S50**		**T**	**L**	**Q**
**2**	18.65	17.52	17.21	19.24	0.409	0.274	0.684	0.072
4	11.79^{b}	12.17^{b}	12.66^{b}	20.96^{a}	1.203	<0.001	<0.001	0.002
8	11.82	10.93	12.65	13.78	0.575	0.385	0.170	0.398
12	11.67^{c}	12.26^{c}	14.48^{b}	19.16^{a}	1.130	0.044	0.012	0.247
24	15.61^{c}	15.85^{c}	16.20^{bc}	20.45^{a}	0.743	0.021	0.046	0.061

a-c values with different superscripts differ significantly at $p \leq 0.05$ among SP treatments. Treatments: S20, S30, S40 and S50 are ~14% CP, SP proportion (% of CP) 20, 30, 40 and 50 respectively; SEM: standard error of the mean; T: Treatment; L: linear; Q: quadratic.
